# Traumatic Atlantoaxial and Fracture-Related Dislocation

**DOI:** 10.1155/2019/5297950

**Published:** 2019-03-18

**Authors:** Carolin Meyer, Peer Eysel, Gregor Stein

**Affiliations:** ^1^Department of Orthopaedics and Trauma Surgery, University Hospital of Cologne, Cologne, Germany; ^2^Department of Orthopaedics, Spine and Trauma Surgery, Helios Klinikum Siegburg, Siegburg, Germany

## Abstract

Traumatic atlantoaxial dislocation due to ligamentous and combined osseous injuries rarely occurs in adults. There are only few cases published in the literature. In this level 4 study, a cohort of nine consecutive patients suffering from traumatic atlantoaxial dislocation has been analyzed regarding morphology of injury, trauma mechanism, and outcome since 2007. Three types of those injuries have been found regarding direction of dislocation indicating the underlying ligamentous injuries as well as the accompanying grade of instability. Firstly, there was rotatory dislocation, if the alar ligaments were injured. Secondly, there occurred horizontal dislocation, when transverse atlantal ligament was damaged additionally. Thirdly, excessive ligamentous injury led to distraction of the atlantoaxial complex resulting in dissociation of the atlas against the axis. Additionally fractures of the atlas as well as of the odontoid process (type II or III according to Anderson/D'Alonzo) were diagnosed frequently. Atlantoaxial dislocation injuries, especially distraction injuries, offer a high risk for accompanied neurovascular disorders deserving reduction followed by surgical fixation. Only rotatory injuries leading to ligamentous damage solitarily can safely be successfully treated conservatively. Understanding of the injuries' morphology is essential, in order to set the correct diagnosis and to implicate the most advantageous treatment regime.

## 1. Introduction

Traumatic atlantoaxial dislocation appears to be an infrequent entity in adults coming along with an outstanding threat to health. To date only a few cases have been published [1–7]. Due to the unique anatomy of the atlantoaxial complex, which provides high-level mobility, it protects and guides the vertebral arteries and the spinal cord. Therefore injuries of these cervical structures are often accompanied by neurovascular complications and can possibly lead to death.

Atlantoaxial dislocation and distraction occur due to ligamentous injuries solitarily or combined ligamentous and osseous lesions.

To date atlantoaxial rotatory and horizontal displacement is usually classified by Fielding or White/Panjabi [8, 9]. Fielding devised four types of these injuries, rotatory injuries, in which C1 uses the odontoid process as center of rotation with no sliding in the horizontal plane is described as type I. Injuries are typed II if the lateral facet joint C1/2 presents the center of rotation and there is a dislocation of three to five millimeters in the anterior-posterior plane. If there is bilateral dislocation and sliding increases up to more than five millimeters in the horizontal plane the lesion is classified as type III; if a dorsal sliding appears with uni- or bilateral dislocation Fielding matches type IV [9]. Differentiating between three types of luxation injuries, White and Panjabi type bilateral anterior displacement of C1 against C2 as type A, bilateral posterior dislocation as type B, rotatory dislocation of the atlas around the ipsilateral facet joint C1/C2 is named type C and rotatory displacement around the contralateral facet joint is called type D. If there is a bilateral displacement around the center of the odontoid process the lesion this is typed E [8]. Most frequently in these cases of rotatory or anterior-posterior dislocation, fractures of the atlas and the odontoid process graded as type II or III according to Anderson and D'Alonzo are diagnosed additionally [1–6].

Distraction injuries resulting in atlantoaxial dissociation require separate observation. These injuries may be accompanied by fractures of atlas and odontoid process as well [4, 6, 10]. As these lesions are supposed to be life-threatening due to affection of the cervical spinal cord and the vertebral arteries, knowledge about the patterns of injury is essential and salvage has to be conducted quickly.

## 2. Materials and Methods

In a retrospective cohort study, nine consecutive patients suffering from traumatic dislocation of C1 and C2 were analyzed, firstly. Trauma mechanism, radiologic imaging, and clinical disorders were examined and main lesions, accompanying vascular or neurological injuries, were assessed. Furthermore treatment and outcome were evaluated. Secondly, the literature was reviewed for English case reports or series describing dislocation of C1 and C2 in adults.

## 3. Results

Trauma imaging and surgical documentation as well as follow-up imaging and electronic health records of nine consecutive patients suffering from atlantoaxial dislocation were analyzed. The patients aged between 24 and 99 years; six patients were female. Regarding main lesions and direction of dislocations three types of ligamentous injuries have been found indicating a growing grade of instability.

Exact results of the cases' analysis can be seen in Table 1.

### 3.1. Rotatory Dislocation of C1 and C2

**Figure 1:**
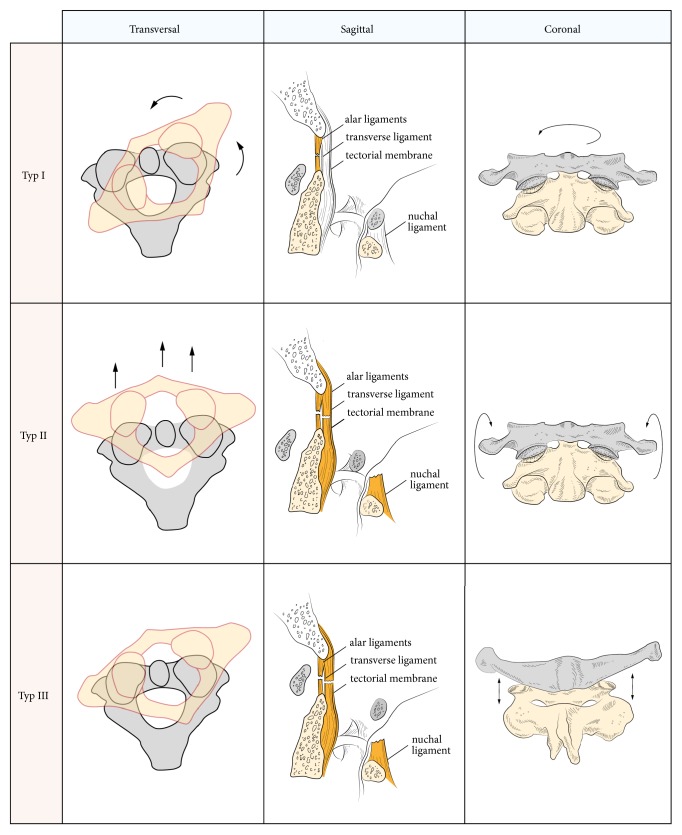
Schematical presentation of the different types of dislocation found in our cohort (transversal, sagittal, and coronal view). Type I represents rotatory dislocation, type II represents an anterior-posterior dislocation, and type III poses a distraction injury. The ligamentous structures, which are damaged, are highlighted in sagittal presentation.

**Figure 2:**
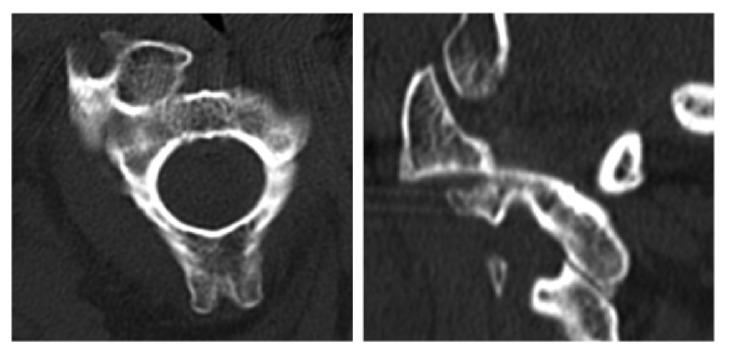
Transversal and sagittal CT imaging of the atlantoaxial facet joints, showing a rotatory injury of the atlantoaxial complex, type II by Fielding [9] demonstrating rotation of C1 around the left atlantoaxial facet joint.

**Figure 3:**
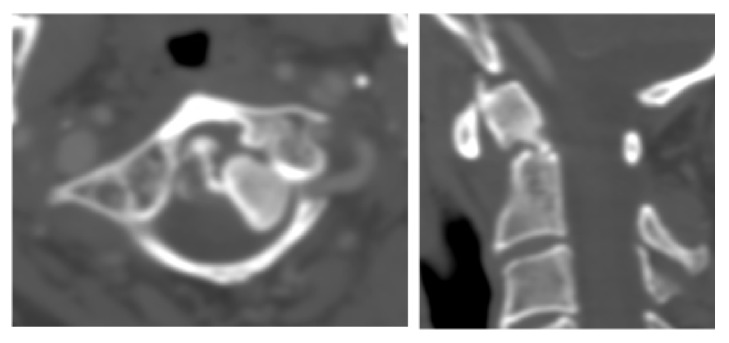
Transversal and sagittal CT imaging of C1 and C2, showing luxation of the left atlantoaxial facet joint (type II according to Fielding [9]) combined with a fracture of the odontoid process (type II according to Anderson and D‘Alonzo).

Three patients, aging 61 to 79 years, showed rotatory dislocation of C1 against C2 solitarily (Figure 1). All of them sustained low-energy trauma as domestic plunge or low stair fall. In one case both lateral atlantoaxial joints were displaced, but in the other two unilateral facet joint dislocation occurred (Figures 2 and 3). The alar ligaments were destroyed in every case as well as the capsule of the dislocated facet joint. Whereas in one patient a sheer fracture of the dislocated facet was found, the other two presented an additional fracture of the odontoid process (Figure 3). One patient, moreover, suffered from a fracture of the atlas, typed II according to Gehweiler, as well as from subaxial lesions. An incomplete paresis of one arm was found during clinical examination. A vascular lesion of the vertebral artery was presented in one case only.

Both patients suffering from atlantoaxial dislocation solitarily were treated by closed reduction successfully. Afterwards, posterior internal fixation was performed using Goel's and Harm's technique [11]. Due to affection of the vertebral artery, medicamentous platelet inhibition was initiated in one of the patients. Follow-up examination showed correct reduction of the atlantoaxial complex and consolidation of the fractures in both patients. Moreover the affection of the vertebral artery resolved completely and platelet inhibition could be stopped after six months.

In the case, where there were subaxial lesions and spinal stenosis leading to neurological disorders, open reduction was performed and occipito-cervico-thoracal fusion, decompression, and additional bone grafting subsequently. Follow-up imaging showed correct reduction but neurological deficits of the left arm bettered only slightly.

### 3.2. Horizontal Dislocation of C1 and C2

**Figure 4:**
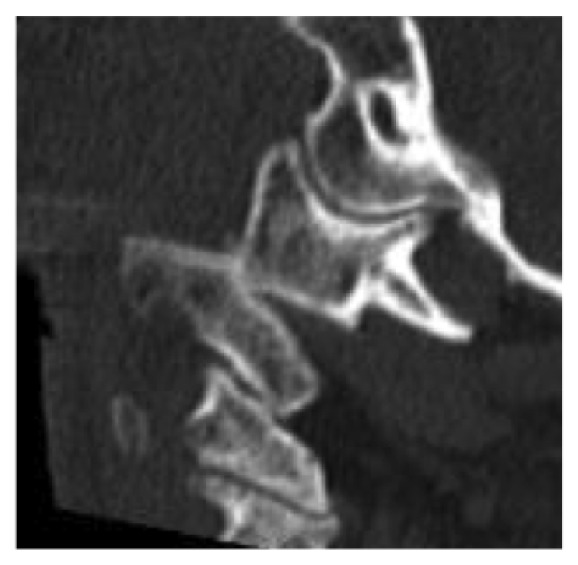
Sagittal CT reconstruction C0 to C3 demonstrating dorsal dislocation of C1 against C2.

Horizontal displacement combined with little rotation of the atlas against the axis was assessed in three patients (Figure 1). Aging from 38 to 99 years, trauma forces appeared to be higher than in solitary rotatory injuries, especially in the younger patients. Alar and apical ligaments as well as the transverse atlantal ligament were damaged and MRI showed different amount of fluid signal at tectorial membrane. In one case, the atlas was found to be displaced ventrally to the axis accompanied by a unilateral sheer fracture at C1. Two patients presented fractures of the odontoid process, typed II according to Anderson and D'Alonzo. In both patients the atlas dislocated dorsally against the axis (Figure 4). In one of these two cases an additional atlas fracture, typed IV according to Gehweiler, was diagnosed as well as large intraspinal hematoma. In this patient, but in none of the others, hemiplegia was examined clinically. The two patients, who were neurologically intact, were treated by closed reduction and posterior internal fixation using the technique described by Harms and Goel [11]. After one year, the implants were removed and the patients stayed neurologically intact. Unfortunately, range of motion was not reported after implant removal. In the third patient, wide decompression was performed at levels C2 and C3 and internal, occipito-cervical fixation was applied afterwards. Nevertheless hemiplegia remained until the patient left the hospital and there was no follow-up.

### 3.3. Distraction of C1 and C2

**Figure 5:**
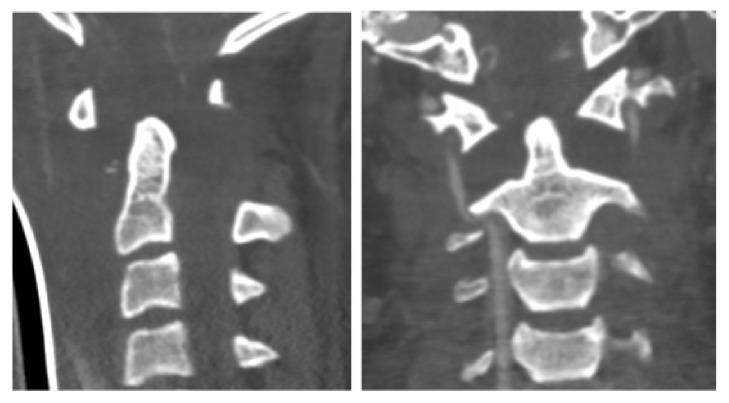
Sagittal and coronal CT reconstruction of C0 to C4, showing traumatic excessive atlantoaxial dissociation.

**Figure 6:**
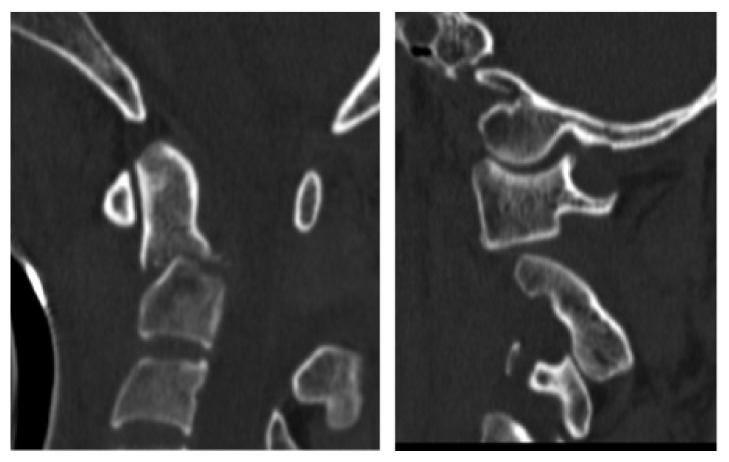
Sagittal CT reconstruction of C0 to C3, showing a low-grade displaced fracture of the odontoid process, classified as type II according to Anderson/ D'Alonzo, combined to a dissociation of C1 and C2.

Distraction injury, which was diagnosed by wide dissociation of the atlantoaxial joints in coronal and sagittal reconstructions of computed tomography, was found in three patients aging 24 to 27 years (Figures 5 and 6). All patients sustained high-energy trauma. Two of them presented an additional fracture of the odontoid process, typed II or III according to Anderson and D'Alonzo (Figure 6). In all cases bilateral dissection of the vertebral arteries was detected. One of them suffered from a spinal shock and died. The other two patients did not show any clinical signs of neurological impairment and were treated surgically. During surgery the very high amount of instability was observed. Open reduction and dorsal fixation using the technique described by Harms and Goel was performed successfully [11]. Correct reduction was pointed out by computed tomography in both cases after surgery and implant removal was performed in one patient. This patient stayed neurologically intact and barely complained about pain during follow-up. The other young patient did not show neurological disorders as well, but did not want to undergo implant removal after consolidation was reported. Affections of the vertebral arteries resolved completely.

## 4. Discussion

Traumatic C1/ C2 dislocation appears to be rare but possibly life-threatening entities of injury coming along with a high risk of vascular and neurological disorders [5, 11–14]. The role of the atlantoaxial structures in trauma is still not completely understood.

Due to the relatively wide extension of the spinal canal at level C1/C2 displacements do not result in neurological disorders necessarily. Distraction injuries, however, may lead to distortion as well as to rupture of the spinal cord depending on the force applied. Accompanying vascular injuries affect the vertebral artery usually, but may involve the carotid arteries as well [10, 13]. Especially patients, who present a ‘high-riding' variant of the vertebral artery, are at risk of vessel dissection or rupture, because the artery is limited obviously due to its guidance provided by the bony structures.

As these types of injuries come along with a high grade of instability causing consequential disorders possibly, understanding of the injuries' morphology is of essential importance, in order to set the correct diagnosis and to apply the appropriate treatment.

Based on the presented, retrospective analysis of patients suffering from traumatic atlantoaxial dislocation we found only three of the four types known, regarding main lesions and direction of dislocation (Figure 1) [14]. Firstly there were rotatory injuries and secondly horizontal injuries and at least distraction injuries were found.

As the alar ligaments are known to be the major restrictor of rotation and lateral flexion, traumatic rotatory luxation appears if the alar ligaments and the facet joint capsules are damaged due to flexion and rotation forces. The transverse atlantal ligament, however, provides rotatory movement of C1 and C2 and remains intact in these cases [15, 16]. Present results agree that rotatory displacements mostly result from low-energy trauma as domestic plunges and that unilateral joint dislocation occurs usually [17, 18]. In the cohort presented, rotatory displacements were mostly accompanied by sheer fractures of the facets and a fracture of the odontoid process. Adams observed similar results in his autopsy series and found solitary rotatory dislocations of C1 against C2 accompanied by ligamentous lesions of the alar ligaments in 14 cases. Additional osseous lesions occurred in some cases. None of the patients showed laceration of the transverse ligament [12].

The transverse atlantal ligament, however, appears to be the main stabilizer of the atlantoaxial complex in the anterior-posterior plane [15, 16]. High-energy, hyperextension trauma, possibly combined with rotatory forces, may lead to horizontal displacement of the lateral facet joints of C1/C2 as well as to disruption of the articulation between the anterior arch of C1 and the odontoid process. In these cases the transverse atlantal ligament, the tectorial membrane, and the anterior longitudinal ligament have to be destroyed besides alar and apical ligaments [16]. Therefore, instability and the risk of accompanied vascular and/or neurological disorders raise [19]. Present analysis agreed that the trauma force leading to such type of dislocation appeared to be higher than in cases, where solitary rotatory displacement was found, especially in younger patients. The transverse atlantal ligament appeared to be the most important factor in the development of horizontal instability. In all cases presented, there was evidence that the tectorial membrane was injured, whereas excessive disrupture of the posterior longitudinal ligament coming along with a large intraspinal hematoma was only observed in one case. The other two patients did not sustain accompanying vascular or neurological disorders.

If nuchal ligaments, tectorial membrane, longitudinal ligaments, lateral atlantoaxial ligament, alar and transverse atlantal ligaments, and the facet joint capsules are damaged by high-energy forces, vertical dissociation of C1 and C2 may occur. Muscles of the neck may be ruptured as well in these cases [16, 19]. This kind of injury occurs if high energy arrives at the head or the neck while the upper cervical spine is inclined and distraction is applied at the same time [12]. Mostly, coronal and sagittal imaging shows an excessively increased distance of the articular surfaces of C1 and C2 as shown in our patients [17].

According to the results presented in the literature, all patients of our cohort, which presented distraction injuries, suffered from disorders of the vertebral arteries and one patient died from spinal shock at least.

Based on recommendations and cases presented in the literature as well as on our present results we conclude that in cases of high-energy trauma as well as in cases of low-energy trauma in older patients it appears to be highly necessary to perform CT imaging after trauma. If atlantoaxial dislocation is diagnosed, angiography is essential. Although direction of atlantoaxial dislocation may lead to the knowledge about all injured structures, MRI has to be performed, in order to visualize injures structures clearly. Moreover, MRI presentation may give evidence about spinal cord injury [13, 20].

Regarding treatment of atlantoaxial dislocation, there is consensus that reduction has to be performed as soon as possible in all cases, in order to prevent residual instability and development of permanent deformity. To date it is known that time to reduction correlates with the recurrence of dislocation recurrence and failure of reduction [14]. Afterwards, immobilization and rigid fixation are necessary due to the high, residual instability. Wise et al. described one case of solitary rotatory atlantoaxial dislocation caused by ligamentous injury, which they treated successfully using brace immobilization [7]. Fielding proposed conservative treatment using brace immobilization for rotatory dislocations of C1 and C2 as well [9]. A close-mesh follow-up, including functional imaging is necessary, in order to prove the treatment's success and to prevent ongoing atlantoaxial instability in the long-term [14, 21].

Most authors, however, prefer surgical stabilization in adult lesions due to the residual instability after reposition of the facet joints, as do we [4, 6, 18, 22]. As rupture or dislocated bony avulsion of the transverse atlantal ligament appears to be clear indicators for the need of surgical stabilization, horizontal atlantoaxial dislocations as well as dissociations have to be treated surgically performing atlantoaxial instrumentation according to the recommendations of Dickman and Kandziora [23, 24]. Moreover it is known that there is a need of surgical intervention, if a fracture of the odontoid process and a rupture of the transverse atlantal ligament are combined [25]. As shown in the present cohort, surgical management appears to come along with good outcome.

Some authors favored performing closed reduction and fixation using a halo fixator. This method, however, failed in three cases. Lenehan et al. published a case in which closed reduction using a halo fixator failed in a patient suffering from C1/C2 lateral dislocation with fracture of the odontoid process [22]. Przybylski et al. reported about a 35-year-old man suffering from vertical atlantoaxial dislocation with additional type III odontoid fracture. The authors pointed out that atlantoaxial dissociation increased in the further course despite stabilization with halo fixator. Therefore they concluded that this method does not eliminate the vertical instability as it boosts it to the contrary [6]. This method, however, is advantageous, if the treatment is delayed or the dislocation is not correctly reduced in the first step [14].

Several methods are known in order to run surgical fixation of C1 and C2. Transarticular fixation using Magerl's method and C1 mass with C2 transpedicular screw fixation according to Goel and Harms are commonly used resulting in satisfying long-term results [11, 26]. Other screw constructs for atlantoaxial fixation include Wright's C1 lateral mass and C2 translaminar screw construct or the C1 lateral mass and C2 pars screw construct [27]. Du et al. performed a meta-analysis of biomechanical testing of these atlantoaxial fixation techniques, which show good to excellent fusion rates in the literature. The authors concluded that all of these fixation techniques provide significant stabilization of axial rotation, flexion, and extension. Instrumentation constructs between lateral masses of C1 and translaminar screw placement at level C2, however, did not provide significant stabilization of lateral bending [28].

From our point of view Goel's and Harms's technique is advantageous in comparison to the transarticular fixation in atlantoaxial dislocation. Firstly, the risk of relapsing dislocation may increase intraoperatively if a screw is inserted as the atlas is possibly pushed away. Secondly, especially in young patients, further violation of the facet joints has to be avoided in order to remove implants after consolidation. Findings of Lee et al. support our experience that C1 mass and C2 transpedicular screw fixation is beneficial compared to transarticular fixation regarding the consolidation rate [29]. In patients presenting with high-riding vertebral artery, however, other fixation techniques are advantageous [28].

If there is a dissection of the vertebral artery, the patient has to get therapeutic anticoagulation by warfarin or phenprocoumon [13].

## 5. Conclusions

Atlantoaxial dislocation is a rare but complex entity of injury. It occurs combined with various combinations of ligamentous and osseous lesions. Only rotatory injuries affecting ligamentous structures solitarily may be successfully treated conservatively, while injuries resulting in horizontal or vertical instability of the atlantoaxial complex require internal fixation of atlas and axis.

## Figures and Tables

**Table 1:** Analysis and classification of the nine cases treated at our institution: osseous injuries, ligamentous injuries, vascular disorders, neurological disorders, and classification.

Cases Sources	A (y)	S	Trauma	Ligamentous injuries	Osseous injuries	Vascular injuries	Neurological injuries	Dislocation	W/P [8]	F [9]
1	61	f	Stumble fall	Apical, alar ligg., right facet joint capsule	Sheer fracture of the right facet of C2			Rotation right lateral facet dislocated	C	2
2	77	m	Domestic plunge	Apical, alar ligg., facet joint capsules	Anderson/ D'Alonzo Type II	Dissection VA one sided	/	Rotation Luxation of both lateral facets	E	1
3	79	f	Low stairfall	Apical, alar ligg., left facet joint capsule	Anderson/ D'Alonzo type II, Gehweiler 3, Tear-drop C3, distraction C 6/7	/	Incom-plete paresis of the right arm	Rotation left lateral facet dislocated	C	2
4	38	f	High stair fall	Apical, alar, transverse ligg., facet joint capsules, tectorial membrane	Sheer fracture of the right facet C1	/	/	ventral	A	3
5	84	m	High stair fall	Apical, alar, transverse ligg., facet joint capsules, tectorial membrane	Anderson/ D'Alonzo type II, Gehweiler 4	Intraspi-nal hematoma	Hemi-plegia	dorsal	B	4
6	99	f	Domestic plunge	Apical, alar, transverse ligg., facet joint capsules, tectorial membrane	Anderson/ D'Alonzo type II	/	/	dorsal	B	4
7	24	f	Motor-cycle accident	Apical, alar, transverse, longitudinal ligg., facet joint capsules	/	Dissection VA bothsided	Spinal shock	cranial	/	/
8	25	f	Car accident	Apical, alar, transverse, longitudinal ligg., facet joint capsules, muscles	Anderson/ D'Alonzo type II	Dissection VA bothsided	/	cranial, ventral	/	/
9	27	m	Motor-cycle accident	Apical, alar, transverse, longitudinal ligg., facet joint capsules, muscles	Anderson/ D'Alonzo type III	Dissection VA bothsided	/	cranial	/	/

## Data Availability

Data used to support the findings of this study are included within the article.
